# Concepts for risk-based surveillance in the field of veterinary medicine and veterinary public health: Review of current approaches

**DOI:** 10.1186/1472-6963-6-20

**Published:** 2006-02-28

**Authors:** Katharina DC Stärk, Gertraud Regula, Jorge Hernandez, Lea Knopf, Klemens Fuchs, Roger S Morris, Peter Davies

**Affiliations:** 1 Federal Veterinary Office, CH-3003 Bern, Switzerland; 2 College of Veterinary Medicine, University of Florida, Gainsville, FL, USA; 3 Institute of Applied Statistics, A-8010 Graz, Austria; 4 EpiCentre, Massey University, Palmerston North, New Zealand; 5 Department of Veterinary Population Medicine, St. Paul, MN 55108, USA

## Abstract

**Background:**

Emerging animal and zoonotic diseases and increasing international trade have resulted in an increased demand for veterinary surveillance systems. However, human and financial resources available to support government veterinary services are becoming more and more limited in many countries world-wide. Intuitively, issues that present higher risks merit higher priority for surveillance resources as investments will yield higher benefit-cost ratios. The rapid rate of acceptance of this core concept of risk-based surveillance has outpaced the development of its theoretical and practical bases.

**Discussion:**

The principal objectives of risk-based veterinary surveillance are to identify surveillance needs to protect the health of livestock and consumers, to set priorities, and to allocate resources effectively and efficiently. An important goal is to achieve a higher benefit-cost ratio with existing or reduced resources. We propose to define risk-based surveillance systems as those that apply risk assessment methods in different steps of traditional surveillance design for early detection and management of diseases or hazards. In risk-based designs, public health, economic and trade consequences of diseases play an important role in selection of diseases or hazards. Furthermore, certain strata of the population of interest have a higher probability to be sampled for detection of diseases or hazards. Evaluation of risk-based surveillance systems shall prove that the efficacy of risk-based systems is equal or higher than traditional systems; however, the efficiency (benefit-cost ratio) shall be higher in risk-based surveillance systems.

**Summary:**

Risk-based surveillance considerations are useful to support both strategic and operational decision making. This article highlights applications of risk-based surveillance systems in the veterinary field including food safety. Examples are provided for risk-based hazard selection, risk-based selection of sampling strata as well as sample size calculation based on risk considerations.

## Background

Emerging animal and zoonotic diseases and increasing international trade have resulted in an increased demand for veterinary surveillance systems, while human and financial resources available to support government veterinary services are becoming more and more limited in many countries world-wide. This constrains all areas of activities of veterinary services, including monitoring and surveillance programmes. One option to respond to this situation is to ensure that the most relevant programmes are maintained. Several government veterinary services, including those of England and Wales (Anonymous, 2000) and New Zealand (Thornton, 2004) have therefore undertaken evaluations of their surveillance programmes. One recommendation of the review in England and Wales was that a structured approach was needed to determine priorities for surveillance, and that this approach should be based on risk (Anonymous, 2000). Consequently, a method to develop risk profiles for animal diseases is being developed (Anonymous, 2003). A similar outcome resulted from the evaluation of surveillance needs in New Zealand, which stated that a 'risk-based' approach to identify priority diseases will provide the basis for resource allocation (Thornton, 2004). These examples illustrate the background and motivation behind the 'risk-based' philosophy for veterinary surveillance. The core rationale underpinning risk-based strategies is that issues that present higher risks merit higher priority for surveillance resources as these investments will yield higher benefit-cost ratios. This axiomatic foundation has led to a rapid rate of acceptance of the concept of risk-based surveillance that has outpaced the development of its theoretical and practical bases. The phrase 'risk-based surveillance' is increasingly prevalent in government documents of many countries, being applied across a range of contexts including chemical residue monitoring, exotic disease surveillance, or sampling for food safety assurance in general.

Inevitably, because the term 'risk' is defined differently in various contexts, its use in relation to surveillance has tended to be vague. The objectives of this article are to review reports on risk-based surveillance, and to develop a framework which may hopefully lead to more uniform use and understanding of both the terminology and its practical implementation.

## Review and discussion

### Terminology

In order to discuss 'risk-based' surveillance, it is essential to clarify the meaning of risk. Risk (as a noun) has numerous common language uses that influence its interpretation. A short list includes 1) the chance of something going wrong; 2) any hazardous entity likely to cause injury, damage, or loss; 3) in insurance, the probability, amount, or type of possible loss incurred and covered by an insurer; 4) in finance, the possibility of loss in an investment or speculation.

Risk has been defined by medical epidemiologists as 'the probability of disease developing in an individual in a specified time interval' (Rothman and Greenland, 1998). In contrast, in the field of risk analysis, risk is defined as the synthesis of two components, namely the probability of occurrence of an undesired event and the consequences or costs of this event (International Animal Health Code). The Society for Risk Analysis defines risk as 'the potential for realization of unwanted, adverse consequences to human life, health, property, or the environment; estimation of risk is usually based on the expected value of the probability of the event occurring times the consequence of the event given that it has occurred.' Thus risk may be viewed as the absolute value of expected losses. We would argue that the epidemiological definition for risk (probability only) is less useful in the context of surveillance than the risk analysis paradigm, because the probability of occurrence of an adverse event alone is not sufficient to drive allocation of resources, and expected consequences of disease events must have substantial influence.

Furthermore, risk is being evaluated in the framework of risk analysis consisting of risk management, risk assessment and risk communication (International Animal Health Code). Surveillance itself is an integral component of both risk management (e.g., as a tool for early detection of an event) and risk communication. The term 'risk-based surveillance' could consequently imply that allocation of surveillance activities is guided by the probability of events with or without consideration of the consequences of the event; the management of the event; or the process of communication (including perceptions) of the event.

The term 'targeted' surveillance has been used to describe concepts which, from an epidemiological perspective, could constitute risk-based surveillance. This term was first used with reference to surveillance programmes of bovine spongiform encephalopathy (BSE) (Doherr et al., 2001, 2002; Morignat et al., 2002). The concept of targeted surveillance for BSE was to concentrate sampling for screening tests on defined sub-populations of cattle that were expected to have a higher prevalence of the disease (Doherr et al., 2001). The sub-populations were defined by age and by so-called 'exit routes', i.e. whether cattle went to normal slaughter, emergency slaughter or were cattle that died or were euthanised at the farm. Similarly, targeted surveillance was defined more generally as surveillance 'focusing the sampling on high-risk populations in which specific, commonly known risk factors exist' under the assumption that the event to be surveyed would be more common in the targeted population than in the general population (Salman et al., 2003). Assuming that the epidemiological intelligence is correct (i.e. high-risk groups can be predicted from risk factors), for a fixed surveillance investment, targeted surveillance should yield both higher sensitivity and higher positive predictive value than surveillance conducted randomly across the population. Martin and Cameron (2003) argue that the assumption in traditional surveillance, that the probability of disease is constant across all individuals in the reference population is not realistic. They propose the evaluation of surveillance systems using scenario trees. The nodes of their trees represent factors affecting the probability of disease occurrence in sub-populations that may be targeted by surveillance. Elsewhere, targeted surveillance was defined as surveillance "to answer a specific question about a defined disease or condition using agreed mechanisms for detection" (Anonymous, 2003), a definition which does not explicitly incorporate risk-based concepts.

In the context of food safety, a 'risk-based food safety system' has been defined by the SaFoodChain working group (SaFoodChain website). This definition states that a risk-based food safety system is 'a system that demonstrates to consumers and other stakeholders that foods are being produced under conditions which minimize adverse health effects. This is achieved by using information on the nature of human health hazards associated with particular food products, the likelihood of consumers being exposed to these hazards, the consequences of exposure and the capacity of the production and processing system to mitigate risks arising from hazards which are above a threshold of concern'. The motivation behind this approach is to promote efficient risk management and to facilitate risk communication, primarily in the context of quality assurance.

### Risk-based approaches in disciplines other than veterinary medicine

Risk-based approaches are widely used in many fields unrelated to animal health. Particularly, ecologists have widely adopted risk-based methods. In the management of environmental pollution risks, the so-called 'risk-based corrective action' (RBCA, 'Rebecca') has become a standard (Standard guide for risk-based corrective action). The formal definition of RBCA is: "A streamlined approach in which exposure and risk assessment practices are integrated with traditional components of the corrective action process to ensure that appropriate and cost-effective remedies are selected, and that limited resources are properly allocated" (EPA, 1995). In RBCA, contaminated sites are classified (scored) according to exposure scenarios and consequences to public health. Corrective actions are then defined for the different site categories. In the categorisation, four tiers of risk assessment were described where the level of detail and complexity of the risk assessment increases from tier I to tier IV ("Use of risk-based decision making").

Modelling approaches have also been used in environmental sciences to predict likely sites of contamination or spills with the objective to target intervention and to obtain cost-effective surveillance (Smalley et al., 2000) or for early warning purposes (Grayman and Males, 2002). Furthermore, environmental scientists developed sampling methods that take into account risk factors for contamination, specifically in water. Such risk-based sampling has been described for heavy metals in water (Preston and Shackelford, 2002), and for watershed monitoring (Foran et al., 2000). Risk-based sampling was also applied using spatial risk factors (Ericson and Gonzalez, 2003).

In the management of both private and government businesses, risk-based auditing is a broadly known concept. This method is primarily used for internal auditing and qualitative scores are used to categorise activities with respect to business risks (McNamee, 1997). Managers are expected to monitor their management practices and processes with special focus on major business risks, e.g. market or financial risks. The motivation for this approach appears to be similar to the motivation behind risk-based surveillance, namely: "The identification, prioritization and sourcing of key organizational risks is critical to ensuring that internal audit resources are allocated to the areas that matter most". Risk-based auditing was also described with relation to auditing of research grants and auditing of subsidy payments.

Closer to the veterinary field, risk-based concepts are applied in the surveillance of human diseases. In Italy, special surveillance programmes were implemented for workers who are exposed to specific occupational risks (Franco et al., 2002). The risk categorisation of workers in this example included both scientific evidence as well as risk perception. The objective of this approach was early detection of disease. Similar concepts are applied in screening programmes focusing on certain age groups, e.g. mammography in women or prostate cancer in men.

### Proposed concepts

In animal health surveillance, decisions at operational or strategic levels can be distinguished. Strategic decisions are needed regarding which issues warrant surveillance, and operational decisions assure the most effective use of resources based on epidemiological principles. One possible framework for terminology, in line with previous authors (Doherr et a., 2001, 2002; Morignat et al., 2002; Salman et al. 2003), would be for the term 'targeted surveillance' to denote use of epidemiological risk factors to optimize population sampling at an operational level. The term 'risk-based surveillance' could be reserved for the context of strategic decision making that employs principles of risk analysis in relation to surveillance of animal disease.

An alternative, that we recommend, is that risk-based surveillance be defined as a more inclusive term in which both the epidemiological and risk analysis perspectives are integrated. Based on the definition of risk and the review of the use of the term 'risk-based' in various fields, we propose to define risk-based veterinary surveillance as follows:

*A surveillance programme in the design of which exposure and risk assessment methods have been applied together with traditional design approaches in order to assure appropriate and cost-effective data collection*.

This relatively broad definition allows for the application of risk assessment approaches at all steps of the design of surveillance systems for early detection and management of diseases or hazards of interest. Table 1 illustrates the different steps during design where surveillance can be based on risk assessment principles. These options for risk-based design will be illustrated in the later sections of this article.

Considering the existing applications of risk-based veterinary surveillance programmes together with applications of risk-based methods in other fields, the objectives of the risk-based approach can be derived. The principal objectives of risk-based veterinary surveillance are:

• to identify surveillance needs to protect the health of livestock and consumers

• to set priorities,

• to allocate resources effectively and efficiently. An important goal is to achieve a higher benefit-cost ratio with existing or reduced resources.

In risk-based systems, public health, economic, and trade consequences of diseases play an important role in selection of diseases or hazards. Evaluation of risk-based surveillance systems shall prove that the efficacy of the risk-based approach is equal or higher than that of traditional surveillance; however, the efficiency (cost-benefit) shall be higher in risk-based systems.

The design of risk-based surveillance systems requires prior, epidemiological knowledge on, e.g., the difference in occurrence of disease between population strata or the influence of risk factors. This type of information cannot be generated by risk-based surveillance systems themselves, but needs to be obtained using traditional, quantitative epidemiological approaches (Table 1).

### Risk-based hazard selection

When surveillance programmes have broad objectives, the list of candidate hazards to be considered can be extensive and selection of hazards becomes critical. For example, the European Union requests that trading partners conduct monitoring of zoonotic pathogens in milk and milk products (Directive 92/46/EEC). The lists of different milk products on the market and potential hazards are very long. However, some agents cannot survive the processing steps employed for specific milk products. For example, Brülisauer et al. (2004) showed that surveillance of zoonotic bacteria in hard cheeses was not cost-effective because the probability of survival of these pathogens through the production process was extremely small. Other surveillance objectives with extensive lists of hazards include surveillance for chemical residues in animal-derived food and the monitoring of antimicrobial resistance in bacteria of animal origin. In both instances, risk-based hazard selection will be required to assure the most important hazards are included. Paige et al. (1999) described how risk profiles were used for substance selection in the national residue monitoring programme in the U.S.A. Ledergerber (2005) used risk profiling and risk ranking to prioritise combinations of bacteria and antimicrobial resistance which should be included in a classification scheme to identify livestock herds that harbour 'important' antimicrobial resistance patterns. For example, fluoroquinolone resistance in Salmonella and cephalosporine resistance in *E. coli *were ranked higher than ampicilline resistance in *E. coli *or Salmonella. This ranking was based on estimated health implications of these resistance patterns when transferred to humans.

The identification and selection of hazards are key steps in the design of surveillance systems, and they will be guided by the objectives of the surveillance. In risk assessment, the equivalent steps are hazard identification and hazard characterisation (Table 1). Consequences of hazard exposure should also be included in the characterisation of a hazard. A limitation in this respect is the lack of information on the nature and size of consequences. In the review of surveillance programmes in England and Wales, it was recommended to use a structured and transparent approach for hazard selection (Anonymous, 2000). Hazard selection was previously conducted with help of decision trees or flow charts in the context of serological surveillance programmes for exotic diseases in pigs in Denmark and Switzerland (Stärk et al., 2000; Hadorn et al., 2002b). The proposed decision tree included hazard characteristics such as prevalence, performance of diagnostic tests, domestic economic implications and international trade implications. Epidemiological data on case frequencies due to specific hazards and results of outbreak investigations provide important input to the hazard identification step.

### Risk-based selection of population strata

Once it has been decided which hazards should be included in a surveillance programme, the next step is the sampling design. In non-risk-based random sampling, all units in a population have the same probability to be selected (Cameron et al., 2003). Limitations of this approach have been identified by several authors. Key concerns are the economic viability of such surveillance programmes when the prevalence of the hazard approaches zero (Paisley, 2001; Doherr and Audigé, 2001; Webb et al., 2001). The cornerstone of risk-based sampling strategies is stratification of the target population into categories that display heterogeneity in probability of harbouring a hazard, or heterogeneity in the severity of consequence if the hazard is present. The probability of individual units within strata to be sampled can nevertheless be calculated, which makes this type of sampling a special case of probability sampling and different from non-probability or purposive sampling as described by Cameron et al. (2003). Strata to be used for risk-based sampling are derived from epidemiological studies assessing the probability of occurrence of the hazard in the stratum (e.g. herds having imported animals from abroad, herds with specific risk factors) and/or the consequence of the occurrence of the hazard in this stratum (e.g. potentially infected animals in sale yards or markets). Prior information on the probability of hazard occurrence in a strata may originate from a range of quantitative epidemiological studies, e.g., risk factor studies. The risk of a stratum as part of a risk-based surveillance design can be derived qualitatively (a risk score or risk category) or quantitatively using risk assessment methods. Results of meta analyses can support this step. Similar to this approach, Thurmond (2003) described a sampling strategy referred to as "proportional risk sampling". In proportional risk sampling, the sample size in different strata reflects the respective risk level.

Unit selection within a risk stratum is done at random and all aspects of random sampling are applicable (Cameron et al., 2003). Issues to be considered in non-risk-based sampling such as test performance and clustering (Martin et al., 1992; Cameron and Baldock, 1998a and 1998b, Ziller et al., 2002) are equally relevant in risk-based sampling.

Many sampling schemes in surveillance programmes that use – at first sight – non-risk-based designs, turn out to use some kind of risk-based strata. For example, samples for laboratory testing are often collected from specified age groups of animals. The reason for this lies in the epidemiology of infectious diseases in populations. For example, older animals are more likely to have antibodies against a hazard (and therefore, to show positive serological results) because they were exposed to a hazard – if it was present. This makes this age group a 'high-probability' group according to our definition. More explicit stratum definitions can be found in protocols for BSE surveillance (see above; Doherr et al., 2001; Morignat et al., 2002).

A more complex approach to stratum definition which takes probability and public health consequence into account to monitor foreign substance levels in imported food of animal origin was described by Breidenbach et al. (2004). They developed a qualitative risk scoring system that took into account the residue legislation and surveillance programmes in the country of origin, the history of residue detection in commodities of the country of origin, the type of commodity (i.e. risk of accumulation of substances), and the public health significance of a substance. Combining these factors, a cumulative, relative risk score was calculated and all substance-commodity-country combinations with the highest scores were included in the programme.

Another way to define risk strata is the incorporation of spatial information. If the underlying phenomena show spatial dependency, point pattern analysis (Diggle, 1983), lattice data (Besage, 1974) or geostatistical methodology (Cressie, 1991) can be used to identify spatial risk factors. Fuchs et al. (2004) applied spatial point pattern analysis to study the resistance behaviour of *Enterococcus *to tetracycline in bulk milk and to identify high density and low density areas. To evaluate space-time interaction of scabies in Styrian chamois, Fuchs and Deutz (2002) used geostatistical methods to find critical distances. Wagner and Fuchs (2005) designed a risk-based sampling plan on the basis of spatial logistic regression for the BHV-1 monitoring in a free region.

### Risk-based sample size calculation

If repeated surveys to document disease freedom are conducted, risk assessment methods can be useful to estimate the likely change in the probability of disease presence since the past survey. In using a risk-based approach, the sample size calculation takes into account the level of confidence that remains from previous survey. The necessary sample size for an upcoming survey is estimated based on the difference to the targeted level of confidence. This approach was proposed by Cannon (2001) and was applied in the context of surveillance to demonstrate freedom from disease by Hadorn et al. (2002a). In this application, a risk assessment was conducted to estimate the probability of hazard introduction (e.g. enzootic bovine leucosis) during a one-year period between two consecutive surveys. Introduction pathways considered were importation of live animals, migratory wildlife or vectors. It was shown that the required sample size for consecutive surveys could be reduced by 60–80 % (Hadorn et al., 2002a, Hadorn et al., 2002b). This reduction in sample size was primarily due to a limited number of live animals being imported from countries with a lower disease status. The accuracy of the estimated sample size does, however, depend on the completeness of the risk assessment. The latter should not only include the probability of hazard introduction but also the consequences of residual, non-detected infection in the country. Additionally, the "aging" of information originating from passed surveys needs to be considered. An improved risk model is being developed to address these issues (Knopf, personal communication).

### Analysis and integration of data from risk-based surveys

Data from risk-based surveillance can be analysed using similar analytical techniques as in non-risk-based surveys (see, for example, Dargatz and Hill, 1996; Audigé and Beckett, 1999; Suess et al., 2002), provided that risk assessment approaches are applied to hazard selection only and extrapolation of results to other hazards is not required. However, when population strata have been selected for sampling based on risk considerations, extrapolation of results to the general population is not possible unless the risk difference between high-risk and low-risk strata is known. This, however, may not be easy to assess. Martin and Cameron (2003) propose the use of scenario trees to compare the sensitivity of random surveillance systems with systems conducted in high-disease-probability populations. They define the *sensitivity ratio *as a measure to assess representativeness *vs*. the level of targeting in a surveillance system.

Furthermore, in international trade, the question of equivalence between sampling designs may become an issue. Trade between countries of equivalent animal health status does not pose a problem, but participating countries need to document their health status. However, when one country is using risk-based sampling and the other is not, they may operate surveillance systems at different confidence levels of case detection. If risk-based surveillance works as expected, a country using the risk-based approach should be more likely to detect positive samples than if using non-risk-based surveillance. This may lead to trade restrictions and other negative consequences. How can the results of one system be compared with the other? If this problem is not resolved, it is likely to pose a major barrier to the adoption of risk-based surveillance in relation to trade.

Another important issue is the integration of data from different sources, risk-based and non-risk-based. Although Cannon (2001) proposed an approach for use of data from different sources, data from risk-based surveillance designs were not considered. One data source may be historical data. The issue of weighting of historic data and the quantification of aging of data are unresolved (Thurmond, 2003). Graphical methods have also been proposed to integrate data from different sources (Hueston and Yoe, 2000), but reports of successful applications of this approach are lacking. In the context of BSE surveillance, where data is being collected from different risk-based population strata, the need of integration of surveillance results became evident (Ducrot et al., 2003).

## Summary

The need for efficient and cost-effective surveillance systems will induce the use of risk-based selection of hazards and population strata, and risk-based sample size calculation. Risk-based surveillance systems offer a more efficient approach for early detection and management of diseases. However, these innovative methods can only be established if there is international agreement on the methodology for risk-based surveillance, and the interpretation of its results. There is an urgent need for the development and evaluation of standardised, internationally accepted methods for risk-based surveillance.

## Competing interests

The author(s) declare that they have no competing interests.

## Authors' contributions

KS, GR, RM and LK developed the proposed concepts for risk-based surveillance. KS and PD were involved in drafting the article. KF contributed aspects on spatial risk. All authors read, critically revised and approved the final manuscript.

## Pre-publication history

The pre-publication history for this paper can be accessed here:



## Figures and Tables

**Table 1:** Steps in the design of veterinary or food safety surveillance programmes, the possible application of risk assessment steps to obtain risk-based surveillance programmes and the epidemiological contributions providing the basis for risk assessments

**Surveillance design steps**	**Risk assessment steps**	**Epidemiological contributions**	**Examples**	**References**
Selection of disease or agent	Hazard identification, hazard characterisation, exposure assessment, consequence assessment	Case reporting, outbreak investigations, systematic review	Selection of diseases based on economic significance for producers, selection of zoonotic agents based on public health significance	Paige et al., 1999; Stärk et al., 2000; Breidenbach et al., 2004; Brülisauer et al., 2004
Sampling				
Selection of strata	Exposure assessment, consequence assessment, risk factors	Risk factor studies, models for population attributable risk, meta analyses	Age strata, spatial strata (regions), product types, products from certain producers	Doherr et al., 2001; Morignat et al., 2002; Breidenbach et al., 2004; Brülisauer et al., 2004
Selection of units	Not applicable (random selection)			
Sample size	Release assessment	Random non-risk-based surveys, cross-sectional studies	Repeated surveys, confidence in disease freedom after defined time periods	Hadorn et al., 2002a
